# Cellular Senescence and Aging in Myotonic Dystrophy

**DOI:** 10.3390/ijms23042339

**Published:** 2022-02-20

**Authors:** Yuhei Hasuike, Hideki Mochizuki, Masayuki Nakamori

**Affiliations:** Department of Neurology, Osaka University Graduate School of Medicine, Suita 565-0871, Osaka, Japan; yhasuike@neurol.med.osaka-u.ac.jp (Y.H.); hmochizuki@neurol.med.osaka-u.ac.jp (H.M.)

**Keywords:** repeat expansion, accelerated aging, alternative splicing

## Abstract

Myotonic dystrophy (DM) is a dominantly inherited multisystemic disorder affecting various organs, such as skeletal muscle, heart, the nervous system, and the eye. Myotonic dystrophy type 1 (DM1) and type 2 (DM2) are caused by expanded CTG and CCTG repeats, respectively. In both forms, the mutant transcripts containing expanded repeats aggregate as nuclear foci and sequester several RNA-binding proteins, resulting in alternative splicing dysregulation. Although certain alternative splicing events are linked to the clinical DM phenotypes, the molecular mechanisms underlying multiple DM symptoms remain unclear. Interestingly, multi-systemic DM manifestations, including muscle weakness, cognitive impairment, cataract, and frontal baldness, resemble premature aging. Furthermore, cellular senescence, a critical contributor to aging, is suggested to play a key role in DM cellular pathophysiology. In particular, several senescence inducers including telomere shortening, mitochondrial dysfunction, and oxidative stress and senescence biomarkers such as cell cycle inhibitors, senescence-associated secretory phenotype, chromatin reorganization, and microRNA have been implicated in DM pathogenesis. In this review, we focus on the clinical similarities between DM and aging, and summarize the involvement of cellular senescence in DM and the potential application of anti-aging DM therapies.

## 1. Introduction

Myotonic dystrophy (DM) is the most common adult form of muscular dystrophy [1]. Patients with DM present with multi-systemic symptoms, including myotonia, muscle weakness, cataract, cognitive impairment, and frontal baldness [2]. In particular, various clinical manifestations of DM, such as muscle wasting, cataract, and hearing loss, share similar characteristics with accelerated aging, implying that aging is involved in the DM process [3]. Hence, understanding the aging mechanisms could lead to a better comprehension of DM pathogenesis. Aging comprises a gradual decline in physiological functions with advancing age. A potential contributor to aging is cellular senescence, defined by permanent cell cycle arrest [4]. Therefore, senescence inducer dysregulation leads to early cellular senescence, potentially contributing to the accelerated aging that characterizes DM. Although accelerated aging-like symptoms in DM have been clinically reported for years, the involvement of cellular senescence in DM pathogenesis remains unclear.

In this review, we describe the common clinical features of DM and accelerated aging. Moreover, we highlight the cellular senescence-related DM pathogenesis and the potential of novel therapeutic strategies targeting senescent cells.

### 1.1. Genetics and Clinical Features of DM

Based on gene mutations, two DM types can be distinguished. Myotonic dystrophy type 1 (DM1) is caused by CTG repeat expansions in the 3′-untranslated region (UTR) of the myotonic dystrophy protein kinase gene (*DMPK*) [5], while myotonic dystrophy type 2 (DM2) is the result of CCTG tetranucleotide expansion in the CCHC-type zinc finger nucleic acid binding protein gene (*CNBP*) intron 1 [6].

Although DM1 and DM2 share major clinical manifestations, such as myotonia, muscle weakness, hearing impairment, and cataracts, certain multi-systemic symptoms, including cognitive dysfunction, daytime sleepiness, primary hypogonadism, and frontal baldness are predominantly DM1-related [7]. Patients with DM1 represent a broad spectrum of clinical severity and symptom onset, and the CTG repeat length correlates with the age of onset [8]. Therefore, based on the time of onset and CTG repeat length, DM1 is classified into mild, classic, childhood/juvenile phenotypes, and it also displays a congenital form (CDM). Above all, patients with classical DM1, the most typical DM1 phenotype, suffer from various systemic symptoms such as cataracts, frontal baldness, sleep disturbance, and fatigue with advancing age, either in parallel with muscle weakness or without skeletal muscle involvement [9,10,11]. CDM is the most severe form of DM, characterized by severe hypotonia and muscle weakness at birth. In adulthood, patients with CDM present classical DM1-like symptoms, including cataracts, frontal baldness, and testicular atrophy [12]. In addition, CDM exhibits developmental delays and intellectual disabilities not observed in other DM1 phenotypes. In contrast to DM1, no congenital form has been found in DM2.

### 1.2. DM Pathogenesis (Spliceopathy)

So far, DM pathogenesis has been characterized as an RNA toxic gain of function due to expanded repeats. Both in DM1 and DM2, the expanded repeats are transcribed into RNA, and the aberrant RNA with the expanded repeats forms foci in the nucleus [13]. RNA containing expanded repeats affect several RNA-binding proteins, including MBNL and CELF1, which regulate alternative splicing. MBNL is depleted by sequestration in the RNA foci [14], while CELF1 is stabilized by phosphorylation [15]. As a result, the dysregulation of these RNA-binding proteins leads to alternative splicing abnormalities. The aberrant splicing contributes to the pathogenicity of DM, referred to as spliceopathy.

### 1.3. Aging and Cellular Senescence

Aging is characterized by a gradual decline in organ function with advancing age. At the cellular level, cells in aging tissue undergo irreversible growth arrest in response to various stimuli. The process is referred to as cellular senescence. Although aging progresses through dynamic and complex biological processes, the excessive accumulation of senescent cells plays a key role in tissue aging [16]. In particular, the accumulation of senescent cells results in tissue atrophy and loss, denervation, hypertrophy, and decreased responsiveness to external stress [17]. The accumulation of senescent cells in local tissues causes progressive functional decline such as osteoarthritis [18], dementia [19], and bone loss [20]. This local dysfunction compromises the healthspan. In addition, the accumulation of senescent cells can affect chronic diseases such as metabolic dysfunction [21], atherosclerosis [22], and cardiac disease [23]. These chronic disorders shorten the lifespan.

Cellular senescence occurs upon various damaging stimuli, such as telomere erosion, oxidative stress, and oncogene activation. First, exposure to these stimuli induces sustained DNA damage. Next, DNA damage, including base lesions, oxidative lesions, single-strand breaks (SSBs), abasic sites, and double-strand breaks (DSBs), activate the DNA damage response (DDR). DDR then promotes H2AX phosphorylation. Finally, it interferes with cell cycle checkpoint inhibitors such as p16 and p53, resulting in cellular senescence [24].

## 2. Common Clinical Features of DM and Aging

**Figure 1:**
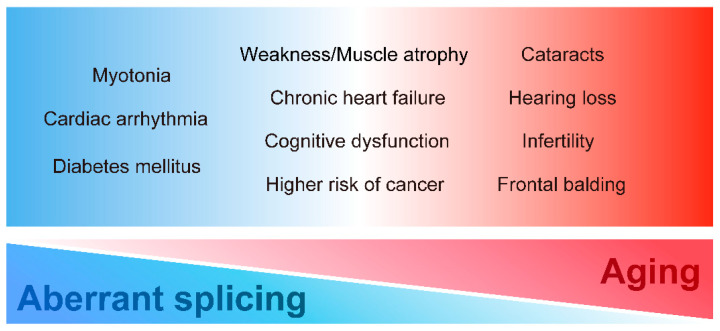
Multi-systemic symptoms in DM. DM has various clinical manifestations in diverse tissues and organs. The symptoms could be closely related to misregulation of alternative splicing, aging, or both.

DM displays various tissue dysfunctions similar to premature aging. In particular, classical DM1 is characterized by multiple aging-related symptoms such as muscle wasting, cognitive dysfunction, and frontal balding. Patients with DM2 also present with aging-related symptoms such as cataracts and hearing loss. Furthermore, these accelerated aging-related dysfunctions in DM are clinically significant. Various age-related symptoms, such as cognitive dysfunction, cataracts, hearing loss, and muscle weakness, interfere with the quality of life and impede social participation, leading to a reduction in the activities of daily living. Given the similarity of symptoms between DM1 and accelerated aging, we propose that premature aging induced by expanded CTG repeat leads to some multi-systemic symptoms of DM in addition to the conventional understanding of DM pathogenesis as spliceopathy. To systematically understand the multi-system manifestations of DM, we summarize each clinical DM feature, especially focusing on DM1, from the premature aging aspect (Figure 1).

### 2.1. Skeletal Muscle Involvement

The primary DM manifestation is skeletal muscle involvement, for example, myotonia, muscle weakness, and muscle atrophy. Myotonia is the most common initial DM symptom, defined by delayed muscle relaxation after voluntary contraction [7]. Nevertheless, patients with DM become less concerned about myotonia as muscle weakness progresses [25]. In fact, unlike most multi-systemic symptoms, the incidence of myotonia decreases with advancing age [26]. The clinical course of myotonia implies its weak relation to aging. Myotonia is reportedly caused by the aberrant splicing of *CLCN1* (predominant exon 7a inclusion) [27] instead of aging.

Patients with classical DM1 present with distal dominant muscle weakness and atrophy, which progresses slowly with advancing age. CDM causes a floppy appearance in newborn infants with hypotonia and severe muscle weakness. In early childhood, children with CDM improve their muscle weakness and acquire the ability to walk; then muscle strength does not deteriorate until adolescence. However, in adulthood, CDM leads to progressive muscle weakness, similar to classical DM1 [12,28]. Here, we focus on the slowly progressive muscle weakness and atrophy commonly seen in adult patients with DM to clarify the aging-related muscle weakness mechanisms. Various studies focusing on mis-splicing have been reported to clarify the pathogenesis of muscle weakness and atrophy in DM. In the previous studies, many splicing misregulation mechanisms of the cytoskeleton- and calcium homeostasis-related genes, such as *DMD* [29], *DTNA* [30], *BIN1* [31], *RYR1* [32], *CACNA1S* [33], and *ATP2A1* [32], have been identified in the DM-affected skeletal muscle. These mis-splicing events are proposed to cause skeletal muscle dysfunction in DM1. However, the aberrant splicing events directly causing progressive muscle atrophy and muscle wasting have not been identified.

Aging-related skeletal muscle involvement includes muscle atrophy and loss of muscle mass and function. The muscle decline is one of the most significant factors of aging-associated changes. The muscle mass, muscle strength, and physical function reduction due to accumulated stress over time are referred to as sarcopenia [34].

Muscle wasting in DM is similar to sarcopenia [35,36]. For example, both disorders could be characterized by a similar distribution of skeletal muscle loss. For example, the atrophy of the temporalis and masseter muscles elicits the characteristic facial appearance of DM1 such as myopathic face or hatchet face [7], one of the most important indicators of sarcopenia [37,38]. Moreover, DM and sarcopenia share histological features such as increased fiber size variation and nucleus internalization [39,40].

Sarcopenia could potentially be caused by the functional impairment of muscle satellite cells, the skeletal muscle stem cells [41]. Although satellite cells are normally quiescent in the adult muscle, they get activated in response to muscle damage, proliferate and differentiate into myoblasts to efficiently regenerate the skeletal muscle [42]. The muscle satellite cell number decreases with advancing age, resulting in impaired muscle regeneration, potentially contributing to sarcopenia pathogenesis [43]. In addition to the age-related decline in satellite cell proliferation, the pathological conditions cause repetitive muscle injury, inducing premature stem cell aging, leading to the reduced proliferative and regenerative capacity of the skeletal muscles [44,45]. For example, Duchenne muscular dystrophy (DMD), a progressive dystrophin mutation-related muscle-wasting disease, increases sarcolemmal membrane fragility and causes muscle damage even under mild stress [46]. Persistent muscle damage due to stress-related vulnerability requires continuous satellite cell regeneration [47]. However, satellite cells fail to meet the increasing demand for regeneration and eventually lose their regeneration ability, leading to muscle atrophy in DMD [47,48].

Although the symptoms and pathogenesis of DMD and DM are not similar, satellite cell dysfunction similar to DMD has been reported as one of the causes of skeletal muscle involvement in DM1. Histopathological distal muscle analysis in DM1 exhibited a two-fold increase in satellite cell numbers and reduced satellite cell proliferative ability in vitro [49]. Moreover, muscle satellite cells in DM1 exhibited premature growth arrest before the exhaustion of their proliferative capacity [50]. These studies suggest that the satellite cell proliferative capacity in DM1 is reduced due to exhaustion secondary to excessive regeneration and premature senescence [49]. Little loss of cell membrane integrity was observed in DM1, unlike DMD [51]. In addition, telomere-independent delayed cell proliferation occurs in DM1 satellite cells [50]. These studies suggest that premature senescence is responsible for satellite cell dysfunction of DM1. The molecular mechanism of premature senescence in DM1 remains to be elucidated, and the potential pathways will be discussed in Section 3.

### 2.2. Cardiac Involvement

The most common cardiac manifestations in DM involve cardiac conduction abnormalities, including atrioventricular block and ventricular arrhythmias, potentially leading to sudden death [52]. Recently, *SCN5A* mis-splicing was identified in the DM-affected heart and is considered to cause cardiac conduction defects [53].

Among other cardiac features, the incidence of chronic heart failure is increased with the progression of DM, although it remains less frequent than arrhythmias [54]. In fact, latent cardiac systolic dysfunction, detected by physiological examination, is relatively common in DM, and exercise limitation due to muscle weakness might mask clinical heart failure symptoms [55]. However, the mechanism behind heart failure in DM remains unknown. Other than *SCN5A* splicing dysregulation, splicing abnormalities, including *DMD* [29], *DTNA* [30], *LDB3* [53], *TNNT2* [56], and *TTN* [57], have been previously reported in DM1-affected hearts, and other splicing misregulations have been identified by RNA-seq analysis [53,58], but no splicing abnormalities have been directly linked to heart failure. Although aging and aberrant splicing seems mutually exclusive, the fact that no mis-splicing event responsible for heart failure in DM has been found does not allow us to conclude that aging is the cause. However, heart failure incidence generally increases with chronological aging, and it is associated with cardiovascular aging, caused by cardiomyocyte and vascular endothelial cell senescence [59]. Despite the low proliferative capacity of the cardiomyocytes, their turnover peaks in childhood and diminishes with age [59]. In particular, telomere length-independent DNA damage due to aging-related mitochondrial dysfunction and increased oxidative stress is reported to induce cardiomyocytes senescence, leading to heart failure [60]. Based on these results, it has been conjectured that premature cardiomyocyte senescence causes heart failure in DM as senescent muscle stem cells lead to muscle wasting, yet the factors causing heart failure in DM remain unclear. Additional aging-related work using DM cardiomyocytes is needed. 

### 2.3. Cognitive Dysfunction

The involvement of the central nervous system in classical DM1 is characterized by cognitive dysfunction, depression, excessive daytime sleepiness, and fatigue [61]. Cognitive impairment is more severe with aging in the case of DM1 than under healthy conditions [62,63,64,65]. Pathologically, neurofibrillary tangles (NFTs) are observed in DM-affected brains, suggesting that DM-related cognitive dysfunction can be regarded as a form of tauopathy [66]. Tauopathy encompasses clinically heterogeneous neurodegenerative disorders, such as Alzheimer’s disease and frontotemporal lobar degeneration, characterized by the brain deposition of the microtubule-associated protein tau, observed as NFTs [67]. In the DM-affected brain, several mis-splicing events have been identified. In particular, aberrant splicing of *MAPT*, encoding microtubule-associated protein tau, potentially causes tauopathy [68,69]. However, the underlying mechanism of how *MAPT* mis-splicing triggers tauopathy remains elusive [70]. Moreover, amyloid β precursor protein *(APP*) is mis-spliced in DM1, but no pathological features could be observed related to the aberrant transcripts [71].

Recent studies also commonly reported tau deposition in autopsies of elderly patients with normal to mild cognitive impairment, namely primary age-related tauopathy (PART) [72], implying the contribution of tauopathy to aging. These observations lead to the hypothesis that cognitive dysfunction in DM may be caused by aging-associated tau accumulation. However, since tauopathy is widely recognized in various neurodegenerative diseases, it remains unclear whether the tau accumulation in the DM brain is due to the aging process. Recent studies have shown that the aging brain also includes senescent cells, and glial cell senescence is particularly involved in aging-related inflammation in the brain [73]. Thus, elucidating the appearances of senescence and inflammation in DM neuron cells will provide insights into the relationship between cognitive dysfunction in DM and aging.

In summary, brain pathology in DM is presumed to be caused by the interaction of RNAopathy, spliceopathy, and tauopathy [74]. Both aging and splicing abnormality-related neurodegeneration could possibly affect the DM-related neurological symptoms.

### 2.4. Endocrine Dysfunction

Endocrine and metabolic dysfunctions, such as diabetes mellitus, hyperparathyroidism, hypertriglyceridemia and thyroid dysfunction, are common in DM1 with advancing age [75,76]. Above all, the increased incidence of diabetes mellitus in DM could result from specific splicing dysregulation. *INSR* splicing abnormalities (exon 11 exclusion), leading to increased insulin resistance were reported in the DM1-affected skeletal muscle [77]. The increased insulin resistance in the skeletal muscle could lead to diabetes mellitus in DM.

On the other hand, aging alters the activities of various endocrine systems through the hormone secretion pattern mainly related to the hypothalamus and the pituitary gland [78]. Interestingly, metabolic dysfunctions in DM are limited to mainly insulin resistance and hypertriglyceridemia, while hypertension, central obesity, and metabolic syndrome are less common in DM1 [79]. Considering that the specific endocrine factors are clinically disturbed in DM, it has been proposed that the cause of endocrine abnormalities is more related to splicing dysregulation than to aging, leading to general hormone secretion changes. Clinically, hormone concentrations vary widely among individuals. In addition, the secretion of hormones is affected by various physiological factors, including inflammation and nutritional status. Hence, it is difficult to elucidate the molecular mechanisms underlying the endocrine disorders of DM. Future studies need to identify DM-specific mis-splicing events in hormone-secreting organs and tissues by removing these confounding factors that affect endocrine function.

### 2.5. Ophthalmologic Abnormalities

Among the ocular manifestations, cataracts occur in more than 50% of the patients with DM1, and are mainly characterized by early onset posterior subcapsular cataract (PSC) [9,80]. In particular, patients with DM suffer from cataracts at an earlier age than those with age-related cataracts [7], and cataracts are occasionally the initial symptom in classical DM1 [9].

The senescence of human lens epithelial cells (HLECs) has been reported to play an important role in age-related cataracts. In general, HLEC reduction is attributed to the declining stem cell proliferative activity within HLECs, namely that of human lens stem cells (HLSCs). In other words, senescent HLSCs cannot replenish new HLECs, and senescent HLECs reside in the lens. The numbers of senescent HLSCs and HLECs increase with advancing age, and PSC severity possibly correlates with the number of senescent HLECs [81]. Furthermore, previous studies using cataract-derived HLECs in patients with DM exhibited reduced cell density and impaired HLEC proliferation [82,83]. These results suggest that HLEC proliferative dysfunction by senescent HLSCs is possibly an underlying mechanism of cataracts in patients with DM. In fact, HLECs in DM1 alter innate immune response- and interferon signaling-related gene expression [84], a feature consistent with senescent cells. In summary, DM comprises a high risk of cataracts, which is potentially attributed to premature senescence-related stem cell depletion.

In addition to cataracts, DM1 reportedly causes changes in the retinal pigment epithelium and the epiretinal membranes, common features of the aging retina, suggesting that DM1 might cause premature retinal aging [85].

### 2.6. Hearing Impairment

Hearing loss is a common symptom in DM1, characterized by sensorineural defects [86,87,88]. Although the hearing impairment mechanism in DM is not sufficiently well understood, transient-evoked otoacoustic emissions, detecting the minute movements of outer hair cells (OHCs) in the cochlea, are markedly impaired in patients with DM, even in patients with healthy hearing, indicating that hearing impairment in DM might be due to OHC dysfunction [87]. In the case of healthy aging, the loss of OHCs is among the most important factors in hearing loss due to the poor OHC regenerative capacity [89]. Although hearing loss in DM has been called “precocious presbyacusis” [90], few studies have examined the involvement of aging in hearing loss in DM. 

No splicing abnormalities causing the auditory disturbance in DM1 have been identified up until now. However, since some abnormalities in alternative splicing have recently been reported as a possible cause of hereditary hearing loss [91,92], splicing dysregulation may also be associated with hearing impairment in DM1. Thus, further studies including aging and/or splicing analyses are needed to elucidate the molecular mechanisms of hearing loss in DM1.

### 2.7. Infertility

Infertility is often a clinical concern in DM, particularly in male patients with DM1 [76]. Patients with DM1 are likely to develop azoospermia due to testicular atrophy and low testosterone levels [93,94,95,96], and sperm motility is inversely proportional to the CTG repeat numbers [97]. Prevalence and determinants of infertility are similar in DM and age-related physiological dysfunction. With advancing age, semen volume and sperm motility decline, and the duration of infertility is prolonged in DM [98].

Meanwhile, research has focused less on female infertility than that occurring in men, being potentially associated with dysmenorrhea, irregular menstruation, and poor response to ovarian stimulation in patients with DM [25,99]. More importantly, female patients with DM exhibit a high risk of pregnancy complications such as miscarriage, premature birth, preeclampsia, and prolonged delivery [100,101]. The preterm delivery of DM might result from CDM effects. However, considering that stillbirth and preeclampsia increase with advancing age [102] and reportedly result from premature uterine and placental senescence [103], these pregnancy-related complications in DM seem to be caused by the maternal aging-like mechanisms. 

In short, the reproductive and perinatal problems occur commonly in both DM and aging, but it remains unclear whether these problems with DM are attributed in part to accelerated aging.

### 2.8. Skin Changes

Patients with DM are well-known to display characteristic frontal balding [2]. In the clinical examination of skin appendages, patients with DM have fine hair with reduced follicular density, similar to androgenetic alopecia (AGA) [104] that is generally closely related to aging. In a previous study using dermal papilla cell (DPC) cultures from balding and non-balding scalps, balding DPCs grew slower in vitro than non-balding DPCs. The reduced proliferative capacity of balding DPCs was linked to senescence-related morphological changes and increased senescence-associated β-galactosidase activity [105]. In addition to frontal balding, several patients with DM also exhibit skin aging, such as severe skin dryness, thin skin, and loss of elasticity [104]. Therefore, skin changes in DM are likely related to aging. 

### 2.9. Higher Risk of Cancer

The increased risk of cancers, including thyroid, uterus, skin, colon, testes, and prostate cancer, has been reported in DM1 [106,107,108,109]. Basically, aging is characterized by cell proliferation suppression, whereas cancer, which is uncontrolled cell growth, increases with age [110]. Although these two concepts seem to be contradictory, advances in cellular senescence research clarified their relationship. While senescent cells suppress tumorigenesis to prevent damaged cell increase, senescent cells promote tumorigenesis by creating a cell-nonautonomous, pro-inflammatory microenvironment [111,112]. The senescence-associated increase in the secretion of pro-inflammatory cytokines, called senescence-associated secretory phenotype (SASP), acts as a potent tumor promoter. Although the activation of various SASP factors is deeply associated with DM (described below in detail), direct evidence that tumorigenesis in DM represents a form of aging is lacking.

### 2.10. Gastrointestinal Symptoms

Gastrointestinal (GI) symptoms are frequent in DM1, especially the upper gastrointestinal tract symptoms including dysphagia, gastric reflux, and dyspepsia, and the lower gastrointestinal tract symptoms including abdominal pain, bloating, diarrhea, and constipation [113,114]. Dysphagia, gastric reflux, and chronic constipation, in addition to the increased incidence of gastrointestinal cancer, are commonly known as aging-related GI symptoms. Although complicated mechanisms including gut bacteria contribute to gastrointestinal aging, recent studies revealed that aging-related changes in individual gastrointestinal cells, such as intestinal neurons, smooth muscle cells, and intestinal epithelial stem cells, lead to GI symptoms [115,116]. However, since the pathomechanism of GI symptoms in DM remains unclear, studies at the level of individual cells in the GI tract are needed to better understand the involvement of aging.

### 2.11. Reduction of Serum IgG Level

IgG is secreted by plasma cells and is involved in humoral immune processes. A decreased serum IgG level has been clinically reported in DM [117]. However, the mechanism of the decrease in IgG is unclear, and the clinical implications caused by the serum IgG decrease in DM, for example, immunological diseases, have not been reported. Changes in IgG glycosylation have been observed with advancing age [118], but no association with DM has been found.

## 3. Splicing Misregulation in DM and Aging

Recently, it has been reported that alternative splicing and its regulation are associated with aging diseases and cellular senescence [119,120]. A previous study using a rat sarcopenia model showed that certain splicing abnormalities, such as *Mbnl1* and *Mbnl2*, were common in DM and aging models [121], indicating the possibility that the mis-splicing in DM might be related to aging. Moreover, previous research suggested that other aberrant splicing events in DM could be associated with aging pathogenesis. The splicing abnormalities of the *CACNA1S*, *RYR1*, and *ATP2A1* genes encoding Ca^2+^ transporters and channels have been identified in DM [32,33]. Such splicing misregulation leads to calcium homeostasis disruption and endoplasmic reticulum (ER) stress signaling activation [122]. Therefore, these mis-splicing events in DM might induce premature senescence through ER stress and be responsible for muscle degeneration in DM [123]. However, aging-related splicing abnormalities, including *TP53, IGF-1, SIRT1,* and *ING1* [124], have not been reported in DM. To date, no studies have shown whether specific splicing abnormalities could directly induce aging. In principle, RNA-binding proteins, such as MBNL and CELF, could affect alternative exons leading to developmental splicing switches. In other words, fetal splicing isoforms are commonly detected in adult DM. It is seemingly illogical that the fetal-type splicing abnormalities could cause aging-like symptoms. These arguments lead to the hypothesis that splicing abnormalities cannot fully explain accelerated aging pathogenesis in DM, and other aging-related mechanisms possibly contribute to DM.

## 4. Relationship between Cellular Senescence and DM Pathogenesis

The main characteristic of accelerated aging is senescent cell accumulation which is triggered by cellular senescence, a state of irreversible cell cycle arrest [125]. Cellular senescence is a complex multistage process, with certain steps implicated in DM pathogenesis. In the following section, we discuss the cellular senescence mechanism in DM to clarify the driver of accelerated aging-like symptoms in DM.

### 4.1. Telomere Shortening

Among the diverse senescence-inducing stimuli, telomere shortening has been particularly known to trigger cellular senescence [126]. Telomeres, the structures at the chromosome ends, cannot be replicated entirely and get shorter with each replication. When telomeres become extremely short, the telomere capping function is lost, and critically short telomeres are recognized as DSBs, leading to DDR [127]. Several studies examining telomere length in DM showed that telomere shortening is accelerated, but senescence is induced before the length reaches the critical size [50,128,129]. These studies indicate that telomere shortening does not directly cause premature cell proliferation arrest, but it is likely to be the consequence of cellular senescence in DM.

### 4.2. Activation of Cell Cycle Inhibitors

DNA-damaging stimuli induce cellular senescence, which is mediated by the cell cycle-regulating tumor suppressor genes. Above all, the representative cell cycle checkpoint genes include *CDKN2A* encoding p16, *CDKN1A* encoding p21, and *TP53* encoding p53 [16]. These proteins are involved in two important cell cycle regulatory pathways, the p53-p21 and p16-pRB pathways. First, the p53-p21 pathway is upregulated upon DDR activation, inducing senescence. p53 regulates p21 expression, then p21 binds to and inactivates cyclin-CDK complexes, resulting in the G1 arrest of the cell cycle [130]. Second, the p16-pRB pathway is upregulated upon sustained stress and maintains senescence. p16 inhibits RB phosphorylation, and unphosphorylated RB binds to the transcription factor E2F1, thereby inhibiting E2F1 release. E2F1 reduction prevents E2F1 target gene transcription, which is responsible for cell cycle regulation [131].

Cells derived from patients with DM might alter cell cycle regulatory protein expression. CDM muscle satellite cells induce p16-dependent premature senescence [50], while DM2 myoblasts trigger p16-independent early growth arrest [128]. These results suggest that cellular senescence occurs in DM1 and DM2 myoblasts, but the mechanism of senescence is different. This difference may be due to the different repeated sequence of DM1 and DM2 or their surrounding sequences. However, how CTG and CCTG repeats affect accelerated aging has not been evaluated so far, and will be clarified in the future by developing cell models expressing different repeat sequences. 

### 4.3. Senescence-Associated Secretory Phenotype (SASP)

Cell cycle-arrested senescent cells in response to various stresses and damages, namely primary senescent cells, can induce senescence by communicating with surrounding non-senescent cells within the tissue microenvironment [111]. This phenomenon is referred to as secondary senescence. Mediators of secondary senescence include cytoplasmic bridges [132], small extracellular vesicles [133], and NOTCH/JAG1 signaling [134]. Furthermore, SASP is the most well-studied mechanism that induces secondary senescence of surrounding cells by secreting signal proteins in a paracrine fashion [135]. These secreted components from senescent cells mainly include pro-inflammatory cytokines such as IL-6, CXCL8 (IL-8), and MCP1 (CCL2). Pro-inflammatory cytokine-related abnormalities have been reported in DM. For instance, CDM myoblasts produce high levels of IL-6 [136], and CDM satellite cells increase PGE2 secretion in vitro [137]. Moreover, NF-κB activation, inducing pro-inflammatory transcription, has been described in DM1 muscle cell [136] and DM1 glial cell [138] models, suggesting that NF-κB might increase SASP in DM. Regarding other SASP components, ECM-remodeling molecules, including MMPs, SERPIN, and TIMPs, reinforce the senescence program [139,140]. DM1 involves both the ECM composition and pro-inflammatory cytokines. Mice expressing expanded CUG repeats display ECM-related gene expression changes [141]. Similarly, we found that DM1 model fibroblasts increase ECM-related gene expression, including *MMP1* and *ADAMTs*, SASP component genes, including *PAI-1*, *IGFBP3,* and induce premature senescence (Hasuike et al., manuscript submitted). These results suggest that the ECM composition imbalance might promote senescence as a part of SASP in DM. In summary, the increased SASP factor secretion strongly affects the cellular senescence in DM.

### 4.4. Mitochondrial Dysfunctions and Reactive Oxygen Species (ROS)

SASP-mediated senescence is also a hallmark of mitochondrial dysfunction, referred to as mitochondrial dysfunction-associated senescence (MiDAS) [142]. Indeed, mitochondria regulate SASP through multiple mechanisms, and morphological and functional mitochondrial changes, such as increased mitochondrial mass and reduced membrane potential, are senescent cell characteristics [143]. A recent study on mitochondria in DM revealed the metabolic disturbance by impaired mitochondria in DM1 fibroblasts, potentially leading to cellular senescence [144]. In addition, in a study using muscle magnetic resonance spectroscopy (MRS), analysis of DM1 skeletal muscle indicated impaired mitochondrial metabolism [145]. 

The dysfunctional mitochondria impair DNA through ROS production and induce cellular senescence [146]. This ROS-mediated mechanism is called oxidative stress-induced senescence [16]. Previous studies showed impaired antioxidant capacity in the serum of patients with DM1 [147,148] and the increased mitochondrial ROS in DM1-affected fibroblasts [144]. Furthermore, ROS and mitochondrial dysfunction contribute to apoptosis induction [149]. Previous studies showed apoptotic pathway activation in DM1 myotubes [150]. Thus, mitochondrial dysfunction observed in DM possibly induces cellular senescence.

### 4.5. Chromatin Reorganization and Nuclear Morphological Change

Chromatin reorganization is a characteristic feature in senescent cells [151]. The most prominent chromatin change of aging is the formation of senescence-associated heterochromatic foci (SAHF) [24]. More importantly, the interaction of chromatin and nuclear lamin plays a key role in aging. For example, nuclear envelope protein lamin B1 (LMNB1) levels are reduced in senescent cells [152]. LMNB1 knockdown promotes heterochromatin rearrangement around the nucleus and SAHF formation [153,154]. Therefore, nuclear membrane composition changes, including that of lamin B1, are among the hallmarks of cellular senescence [152,155]. Clinically, certain progeroid disorders are caused by mutations in nuclear envelope proteins, leading to nuclear envelope structure disruption [3]. Similar to the progeroid syndromes, DM1 displays nuclear envelope organization changes. Fibroblasts of patients with DM1 alter emerin, as well as lamin A/C and B localization, and the size and shape of the nuclei change accordingly [156]. Furthermore, lamin B1 downregulation occurs specifically in DM1-affected myoblasts, suggesting an effect on cell senescence [3]. DM2-affected myoblasts accumulate heterochromatin, a morphological feature of senescent cells [157]. Thus, nuclear membrane organization and chromatin changes, common characteristics of senescent cells, may be involved in the DM phenotype resembling accelerated aging.

### 4.6. MicroRNA

MicroRNA (miRNA), a short non-coding RNA, is an important post-transcriptional regulator of cellular senescence [158,159]. Various miRNAs show dynamic expression changes with advancing age in multiple organs and tissues. In particular, some miRNAs play an important role in the above-mentioned two major senescence pathways, the p53-p21 and p16-pRB pathways, and regulate cellular senescence [158].

Similar to aging, several miRNAs are differentially expressed in the serum and skeletal and cardiac muscle in DM [160,161,162]. Interestingly, we have found evidence for expression changes of senescence-related miRNAs such as miR-152 and miR-15a, in a DM1 cell model (Hasuike and Nakamori, unpublished data). Since individual miRNA broadly regulates cellular senescence by targeting multiple genes and pathways [163], miRNAs might be key modulators in DM-related cellular senescence.

## 5. Therapeutic Potential Targeting Cellular Senescence in DM

Based on an RNA toxic gain of function, considered as a major DM pathogenic mechanism, the treatment targeting toxic RNA has been approached in several ways [164]. Although about twenty drugs for DM1 in categories of small molecules, oligonucleotide-based therapies, and gene therapies have shown promising results in preclinical models or human clinical trials [165], no curative treatment is available in patients with DM at present.

In addition to DM1-specific therapies, anti-aging agents are also promising candidates for the treatment of DM1, as some agents have already been shown to be safe in humans and can be applied to clinical practice rapidly. As described above, accelerated aging is deeply related to the clinical DM features, but no DM-related treatment focusing on cellular senescence has yet been developed. In general, eliminating senescent cells is beneficial, and novel strategies for removing senescent cells without genetic manipulation are attracting attention [4]. Here, we discuss the therapeutic targets of aging and the potential applications in DM.

Two major approaches have been proposed for cellular senescence treatment: senolytics and senomorphics [166]. Senolytics is a treatment specifically eliminating senescent cells through apoptosis. For example, the BCL-2 family regulates cell death through apoptosis and autophagy, and targeting its activity allows apoptosis initiation in senescent cells [167,168]. In addition, the p53-p21 pathway is a promising anti-aging target. Inhibiting the interaction between p53 and FOXO4 induces intrinsic cell apoptosis, and the administration of a peptide interfering with FOXO4 and p53 binding improves senescence [169]. Moreover, p21 knockdown induces cell death in senescent cells [170].

Senomorphics involves a treatment inhibiting SASP secretion without altering cell viability. For instance, rapamycin, an mTOR inhibitor, reduces SASP via the NF-κB transcriptional activity inhibition and extends mouse lifespan [171,172]. Metformin inhibits NF-κB migration to the nucleus, limiting its transcriptional activity [173]. These drugs have been demonstrated to exhibit certain therapeutic effects in both a DM mouse model and cells. Indeed, rapamycin improves muscle relaxation and increases muscle strength in DM model mice without affecting the splicing regulation [174]. Metformin restores mitochondrial dysfunction and cellular senescence in DM1 fibroblasts [144], and improves the exercise capacity and walking ability of patients with DM [175]. Therefore, treatments targeting cellular senescence might be effective in DM and propose promising novel targets for DM therapy.

## 6. Conclusions

**Figure 2:**
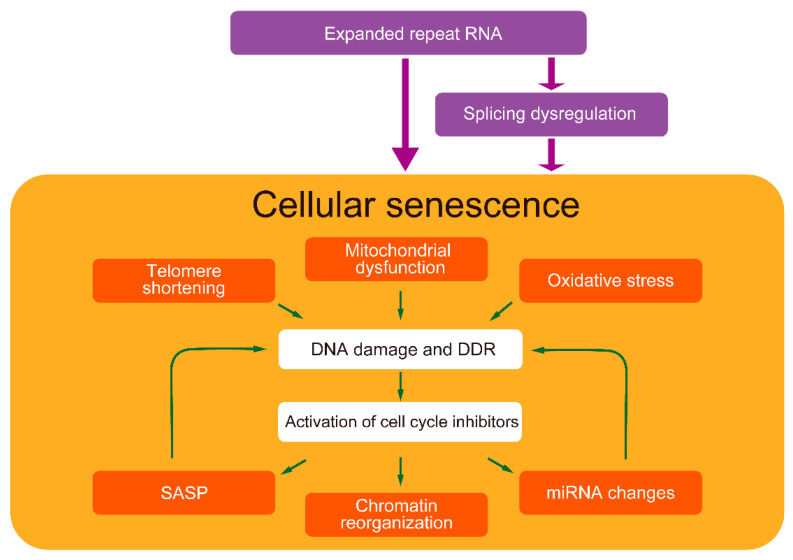
Cellular senescence-related pathways in DM pathogenesis. Expanded repeat RNA possibly induces cellular senescence partly directly and partly through splicing dysregulation. DM exhibits various cellular senescence features. Senescence-inducers, such as telomere shortening, mitochondrial dysfunction, and oxidative stress, cause DNA damage and DDR, activating cell cycle inhibitors, leading to cellular senescence. These factors have been identified in the cells of patients with DM and model cells expressing expanded repeat RNA. In addition, DM exhibits senescent cell features such as SASP, chromatin reorganization, and miRNA changes. SASP and miRNAs might also induce cellular senescence. Splicing dysregulations, a major DM pathogenesis, might also be related to accelerated aging.

Among the multi-systemic DM symptoms, especially muscle weakness, cognitive decline, cataracts, infertility, and hearing impairment are similar to those observed in premature aging. In addition to splicing dysregulation, cellular senescence might contribute to the pathological process leading to various multi-systemic symptoms in DM. Cellular senescence is regulated interactively by multiple cellular stresses [176]. In particular, the positive-feedback loop of mitochondrial damage, ROS production, and DDR activation via the p53-p21 pathway is important for maintaining senescence [177]. In addition, SASP promotes senescence through the autocrine positive-feedback loop [24]. Since certain feedback loop components have also been found in DM, complex senescence regulation might be involved in DM pathogenesis (Figure 2). Although the underlying mechanism of how expanded CUG repeats cause cellular senescence is not fully elucidated, understanding the senescence mechanism in DM could potentially provide new therapeutic targets.
